# Towards XNA nanotechnology: new materials from synthetic genetic polymers

**DOI:** 10.1016/j.tibtech.2014.03.010

**Published:** 2014-06

**Authors:** Vitor B. Pinheiro, Philipp Holliger

**Affiliations:** MRC Laboratory of Molecular Biology, Francis Crick Avenue, Cambridge, CB2 0QH, UK

## Abstract

Nucleic acids display remarkable properties beyond information storage and propagation. The well-understood base pairing rules have enabled nucleic acids to be assembled into nanostructures of ever increasing complexity. Although nanostructures can be constructed using other building blocks, including peptides and lipids, it is the capacity to evolve that sets nucleic acids apart from all other nanoscale building materials. Nonetheless, the poor chemical and biological stability of DNA and RNA constrain their applications. Recent advances in nucleic acid chemistry and polymerase engineering enable the synthesis, replication, and evolution of a range of synthetic genetic polymers (XNAs) with improved chemical and biological stability. We discuss the impact of this technology on the generation of XNA ligands, enzymes, and nanostructures with tailor-made chemistry.

## Highlights

• We review recent advances in nucleic acid chemistry and polymerase engineering that have enabled the synthesis, replication, and evolution of a wide range of nucleic acid-like synthetic genetic polymers (XNAs) with improved chemical and biological stability.
• We discuss the likely biotechnological impact of the further development of XNA technology for the generation of novel ligands, enzymes, and nanostructures with tailor-made chemistry.

## Nucleic acids are versatile materials

Natural nucleic acids, DNA and RNA, are uniquely suited to their information storage role as they allow accurate propagation of chemical information through specific base pairing during replication. DNA and RNA are composed of a limited set of four chemically analogous monomers – the nucleosides adenosine, guanosine, cytidine, and thymidine (or uridine for RNA). The natural nucleosides are composed of three chemical moieties – an aromatic nucleobase, a conformationally flexible ribofuranose, and the anionic phosphate diester linkage between monomers. Nucleic acids can thus be viewed as aperiodic polymers composed of these tripartite nucleotide building blocks.

Although all three moieties contribute to the physicochemical properties of the final nucleic acid polymer, there is a dominant contribution by the polyanionic phosphodiester backbone [1], which decouples the physicochemical properties of a nucleic acid from its information content (i.e., its sequence). Thus all nucleic acid sequences display broadly similar physicochemical properties in sharp contrast to proteins, where a single mutation can lead to radical changes in physicochemical properties like solubility.

The polyanionic nature of the backbone also favours an extended conformation in low ionic conditions through charge repulsion. Nevertheless, single-stranded DNA and RNA can both fold into defined structures, which allow nucleic acid ligands (aptamers) and catalysts (nucleic acid enzymes, NAzymes) [2] to be developed. Furthermore, multiple strands can be designed to associate in defined ways allowing complex 3D objects to be assembled [3,4].

We focus on some of the recent advances in nucleic acid-based materials, particularly enzymes and nanostructures, and on the new possibilities that synthetic genetic polymers can bring to the field.

## Evolution is the greatest asset of nucleic acid-based materials

It is challenging to develop complex function from conventional polymers because their rational design imposes huge demands: understanding of the molecular basis of the desired function, knowledge of the polymer molecular structure required for function, and tight control of polymer synthesis with the desired structure. These challenges remain largely unresolved, but significant progress has been made not only in nucleic acids, but also in peptides and proteins [5–8].

The capacity for replication sets nucleic acids apart from other polymers: replication enables evolution and the iterative optimisation of function. Cycles of sequence diversification and purifying selection enable the systematic search of vast populations (typically 10^14^ variants in nucleic acid selections) for complex phenotypes, such as ligand binding and catalysis, bypassing current limitations of rational design approaches.

**Figure 1:**
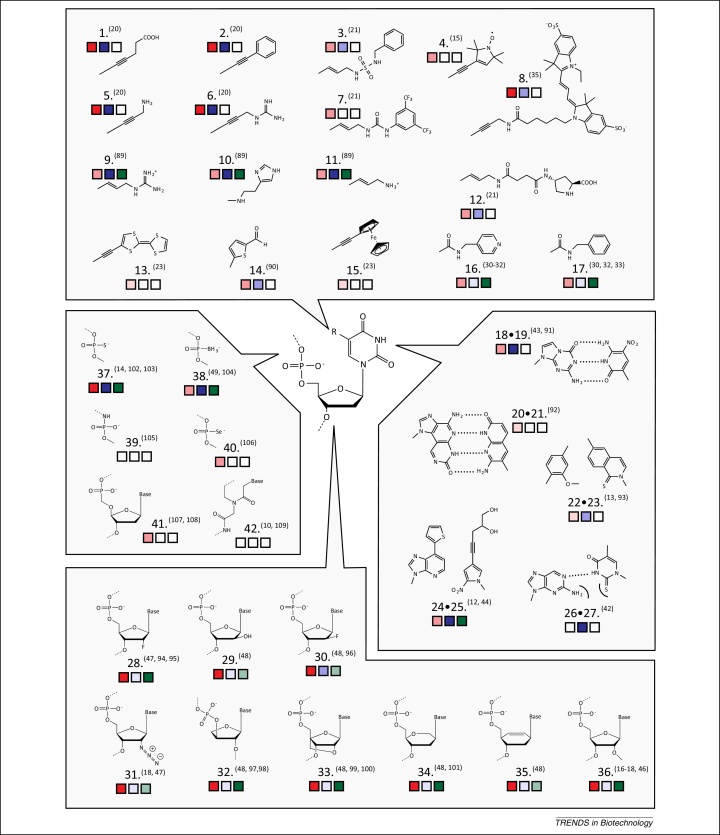
The potential of nucleic acid-like synthetic genetic polymers (XNA) as polymerase substrates, genetic materials, and functional nucleic acids. Each modification is scored for its potential as a polymerase (natural or engineered) substrate as monomers (red) or as templates (blue). Modifications are also scored for their highest reported functional complexity (green). Nucleotide triphosphates or the closest suitable analogue were scored as: , no reported incorporation or extension; , single or sparse incorporations; , multiple incorporations or partial substitution; , full substitution. The suitability of XNAs as templates are scored as: , no reported replication; , DNA synthesis from XNA template; , XNA synthesis from DNA/XNA hybrid template; , XNA replication. Functional complexity was scored as: , no proven function; , functional genetic material; , functional ligands or enzymes. The relevant references are indicated by the XNA shown [10,12–18,20,21,23,30–33,35,42–44,47–49,89–109].

The natural nucleic acid polymers (DNA and RNA), however, have limited chemical diversity and poor chemical and biological stability, limiting their suitability for many applications. Although many modifications of the natural chemical moieties or even introduction of novel chemical functionalities are possible, the challenge is that these are frequently incompatible with replication and hence evolution (Figure 1).

In nature, nucleic acid replication is carried out by complex, highly dynamic enzymes called polymerases, through the sequential incorporation of activated monomers (nucleotide triphosphates) against a single strand of nucleic acid template. DNA polymerases have evolved stringent specificity mechanisms to exclude noncognate substrates from their active site and, as a result, the range of the chemical modifications that can be incorporated and/or replicated with DNA polymerases is limited [9]. Chemical replication is possible but despite significant recent advances [10,11], it remains relatively inefficient imposing limits on the fidelity and polymer length that can be maintained.

Recent research has therefore focused on identifying strategies to circumvent the restrictions imposed by the narrow polymerase substrate spectrum. These efforts can be broadly summarised by three complementary strategies: the chemical optimisation of unnatural substrates improving their uptake by natural polymerases [12,13], the direct engineering of the polymerases to expand their substrate range [14–18], and the use of alternative nucleic acid processing enzymes (e.g., ligases) to access alternative routes to nucleic acid synthesis [19]. All three strategies have proven successful, enabling the synthesis (and to a lesser degree replication) of functional nucleic acid polymers bearing varying degrees of modification.

## When natural is not enough: DNA and RNA as scaffolds

Systematic exploration of the chemical neighbourhood of natural nucleotides has identified a significant number of modifications that are well tolerated by natural polymerases, notably: substitutions to N7 in purines and C5 in pyrimidines (that place the substituent in the sterically unconstrained major groove face of the double helix), unnatural base pairs (that retain or mimic the geometric shape of the cognate pairs), and a few phosphate backbone modifications (that retain the negative charge). These optimised substrates can be incorporated efficiently as partial substitutions, either as a mixture with the natural substrate for very low density labelling or with one of the nucleotides fully replaced by the modified counterpart.

A number of small non-fluorescent substitutions identified by Famulok and colleagues could even be introduced at ‘full’ substitution (all four bases), and allowed direct amplification (by PCR) of the modified nucleic acid, resulting in a double-stranded polymer with significantly altered structural and physicochemical properties (fDNA) [20]. A much wider range of chemical functionalities have been added to natural nucleobases and incorporated by natural polymerases with full replacement of a single nucleotide including amino acids (e.g., proline [21] or tyrosine [22]), redox active groups [23], complexation ligands [24], and many others with a view to improving the catalytic activity of NAzymes.

Although DNAzymes with RNAse activity had been previously reported [25], Joyce, Barbas, and colleagues were the first to demonstrate that functional nucleic acids could be selected harbouring modified nucleobases [26]. They replaced thymidine with deoxyuridine harbouring an imidazole moiety attached to C5 and isolated a Zn^2+^-dependent DNAzyme with RNAse activity. Such modifications increase the chemical diversity in nucleic acids and have been further explored by Perrin and colleagues, who identified other possible nucleobase substitutions to generate DNAzymes, harbouring two (5-aminoallyl-dUTP and 8-histaminyl-dATP) [27,28] or three (5-guanidinoallyl-dUTP, 5-aminoallyl-dCTP, and 8-histaminyl-dATP) [29] fully substituted nucleobases, that are capable of efficient RNA cleavage. The addition of protein side chain-like chemical moieties to the nucleobases allowed the isolation of enzymes that could achieve high catalytic rates in the absence of divalent cations and in physiological-like conditions.

Eaton and colleagues successfully introduced methyl pyridyl modifications to the C5 position of uridine through a rigid carboxamide linker that is well tolerated by natural RNA polymerases and reverse transcriptases [30]. The pyridyl moiety was chosen for its diversity – it could provide hydrogen bonding interactions, hydrophobic or dipolar interactions, and metal coordination sites that are not naturally present in RNA. RNAzyme alderases were successfully selected (harbouring all uridines replaced with the modified nucleobase) and dependent on the pyridyl moieties in the reaction conditions tested [31].

Other chemical functionalities have been introduced to deoxyuridine through a similar carboxamide linker and deployed particularly in the selection of aptamers [30,32,33]. Work by Eaton, Janjic, and their colleagues show that hydrophobic moieties can significantly enhance the binding affinity of the isolated nucleic acid ligands [32,33]. These data suggest that a lack of appropriate hydrophobic contacts may be one of the reasons for failures to isolate high affinity nucleic acid ligands to some protein targets. In a proof-of-principle aptamer against platelet-derived growth factor B (PDGF-BB) from a random library containing benzyl-dU, the chemical neighbourhood of each modified nucleotide in the isolated aptamer was systematically screened to optimise binding affinities [33]. Although the benzyl modifications provided large gains over the unmodified sequence, chemical optimisation of the substituents improved aptamer affinity only modestly. Notably, natural nucleic acid aptamers against PDGF-BB already have a very high affinity (∼28 pM [34]) and thus the potential benefits of the hydrophobic substituents and their optimisation are probably underestimated in this study.

Polymerase engineering also allows more diverse modifications to be accessed, such as fluorescent dye-labelled nucleotides, with applications in next-generation sequencing, arrays, and fluorescent *in situ* hybridisation (FISH). The steric bulk of the large hydrophobic dyes limits their incorporation by polymerases to sparse incorporations. However, directed evolution through compartmentalised self-replication [35] isolated a polymerase variant capable of PCR amplification of double-stranded DNA fragments with full substitution of dCTP with the widely used Cy3- or Cy5-labelled-dCTPs. The resulting ‘CyDNA’ is highly fluorescent and displays significantly altered physicochemical properties including organic phase partition and an up to 40% increased diameter as judged by atomic force microscopy. Mixed Cy3–Cy5 incorporation also converts CyDNA into a photoswitchable biopolymer [36] that can be exploited in applications that rely on quenching, photoswitching, excimer fluorescence enhancement, and chromatic shift, among other photophysical properties – similar to properties observed by Kool and colleagues for synthetic arrays of C-nucleosides [37]. CyDNA, fDNA, and other modified nucleic acid polymers [38–41] provide a first glimpse of the new types of nanomaterials accessible through the ordered display of chemical functionalities on the nucleic acid scaffold and the novel physicochemical properties they engender.

Extended efforts by the Benner [42,43], Romesberg [13], and Hirao [12] groups have yielded a number of unnatural base pairs that are incorporated and replicated with efficiencies approaching the natural bases. In the future, these should allow expansion of the genetic alphabet and enable sparse and precise placement of a wide range of chemical functionalities enhancing the functional potential of DNA aptamers, enzymes, and nanostructures. In a proof-of-concept study, Hirao and colleagues have recently reported the successful isolation of picomolar affinity aptamers against vascular endothelial cell growth factor-165 (VEGF-165) and interferon-γ harbouring the unnatural hydrophobic base 7-(2-thienyl)imidazole[4,5-b]pyridine (Ds) [44].

## When natural is not enough: new backbones

Chemical modification of nucleobases does not alter the nucleic acid backbone and therefore generally does not greatly improve resistance to degradation by exo- and endo-nucleases, or the pH-dependent decomposition of the polymers. By contrast, modification of the nucleic acid backbone can dramatically alter its chemical and biological stability. However, until recently, efficient enzymatic synthesis of synthetic nucleic acid polymers (XNAs) with fully altered backbone chemistries had not been possible.

Partial substitution, through the incomplete replacement of the natural nucleic acid backbone with synthetic alternatives, provides significant gains in stability and has been used to isolate aptamers and RNAzymes with increased resistance to nuclease degradation. Beigelman and colleagues substituted pyrimidine ribonucleotides for 2′-amino-2′-deoxyribonucleotides to select an RNAzyme with RNA nuclease activity [45]. The resulting RNAzyme (1–9t) had high catalytic efficiency (*k*_cat_/*K*_m_ = 3.7 × 10^6^/M/min) at physiological conditions and was stable in human serum (*t*_½_ = 16 h, cf*. t*_½_ < 1 min for natural nucleic acids in serum).

Notably, pioneering work on the rational design of RNA polymerases by Padilla and Sousa [18] identified polymerases capable of synthesising nearly fully substituted XNAs with ribonucleotides harbouring 2′-modified sugars that enabled the selection of 2′-O-methyl-DNA (2′OMe-DNA) anti-VEGF aptamers [46]. Brakmann and colleagues have combined rational design with screening strategies to isolate a T7 mutant capable of efficient, fully substituted 2′-OMe-DNA synthesis [17].

Directed evolution and rational design of DNA polymerases has recently enabled our lab to synthesise and replicate eight different XNAs in which the canonical five-membered ribofuranose ring of DNA and RNA is replaced by various congeners, including hexitol nucleic acids (HNA), cyclohexenyl nucleic acids (CeNA), locked nucleic acids (LNA), threose nucleic acids (TNA), arabinonucleic acids (ANA), fluoroarabinonucleic acids (FANA), 2′-azido-2-deoxyribonucleic acids, and 2′-fluoro-2′-deoxyribonucleic acids [47,48]. These show a whole range of new and interesting properties ranging from exceptional strength of hybridisation (LNA) to complete resistance to degradation by nucleases or acidic pH (HNA). This greatly extends the accessible nucleic acid chemistries that can be used to design and evolve new materials – and these might benefit from, among other properties, increased stability enabling robust re-usable diagnostics, therapeutics, and nanomaterials.

Modifications of the phosphodiester backbone itself are also possible, but provide an even bigger challenge for polymerase engineering. Conservative replacement of one of the non-bridging α-oxygens with sulfur (phosphorothioates) or borano (boranophosphates) moieties has been shown to allow synthesis of the modified nucleic acid at full replacement [14,49,50]. However, these modifications have only modest effects on the backbone charge and conformation. Enzymatic replication of more radical backbone modifications, such as the uncharged peptide nucleic acid (PNA) backbone, are likely to be very challenging to implement. In these cases, non-enzymatic synthesis strategies may be an attractive alternative. Liu and colleagues have developed multiple display systems based on the chemical synthesis of PNA molecules against a DNA template [10,19,51]. Activated PNA pentamers are used as chemical building blocks and result in either a PNA molecule being displayed or a known sequence polymer if the PNA is used as a scaffolding molecule. This enables functional selection of the synthesised polymer, while still covalently attached to its original DNA template [51].

## Nucleic acid nanotechnology: XNAs on the fringe

Due to the programmable nature of interactions through Watson–Crick base pairing, nucleic acids are an attractive substrate for the construction of a wide variety of 2D and 3D nanostructures and devices.

Broadly, there are three strategies currently available for the design and assembly of DNA nanostructures: rational-designed DNA oligonucleotides, DNA origami, and DNA bricks.

Seeman and colleagues pioneered the rational design of oligonucleotides to assemble DNA nanostructures [52,53]. Oligonucleotides are designed to maximise the stability of the DNA device while minimising unwanted interactions, allowing structures to be assembled sequentially and open to further engineering [54,55]. Modified nucleotides can be incorporated in the design to allow the display of increased chemical functionality or as anchor points for further derivatisation [56–58].

DNA origami consists of using short oligos (‘staples’) to guide the folding of a long (‘scaffold’) strand. It was originally developed by Rothemund [59] and refined independently by Kjerms, Shih, and their colleagues allowing complex 2D and 3D structures to be assembled [60,61] – including curved nanostructures [3].

Complex nanostructures such as gated transmembrane channels have been assembled by DNA origami, highlighting the potential of the method [62,63]. In both cases, nucleic acid modifications were introduced to facilitate the structure interaction with membrane bilayers. Dietz, Simmel, and colleagues, using a nanostructure reminiscent of the α-haemolysin transmembrane channel, introduced cholesterol modifications in key staple oligos that were sufficient to drive correct positioning of the channel on the membrane [62]. Using a different pore architecture, Howorka and colleagues introduced phosphorothioate patches to the nanopore design that could be post-synthetically alkylated, creating a ring of neutral backbone, which was sufficient to position the DNA structure on a lipid bilayer [63].

Recently, Yin and colleagues have reported a third approach for the design of complex 3D DNA structures based on the assembly of short oligonucleotides (without a scaffolding strand) termed DNA bricks [64]. The key to this approach lies in linking structural units (8-mers) to physical location, in effect creating an eight-nucleobase address for each position in a discrete 3D space. By ensuring that all addresses are unique, Yin and colleagues made it possible to assemble 3D structures based on predesigned sets of short DNA oligomers (‘bricks’) – each DNA brick a 32-mer, designed as four contiguous eight-base domains targeting adjacent physical addresses, reminiscent of a 2 × 1 LEGO^®^ brick.

DNA nanostructure conformation and assembly can be controlled by the addition of extra DNA strands (DNA fuel) [65,66] or other external stimuli [67,68]. Sleiman and colleagues have refined the design and assembly of DNA polyhedra and nanotubes developing nanostructures capable of switching conformation in the presence of external stimuli [69,70]. They also demonstrated that these nanotubes can be engineered to encapsulate a defined cargo and trigger its release upon conformational change [71]. DNA structures generated by DNA origami are also subject to such control and may be interfaced with other DNA technologies, such as DNA computing [72].

Correct folding can be a limiting factor in the assembly of large DNA nanostructures. Although simple polyhedra can achieve correct assembly yields as high as 90% [54], one-pot assembly of complex structures can vary considerably but is rarely high-yielding [61,64] even after optimisation of folding conditions. Capping oligos, which contribute to the structure as well as providing a short external single-stranded segment of unstructured DNA (i.e., a poly-dT homopolymer) have been used to increase the efficiency of correct assembly [60,64] by minimising interstructure contacts.

Investigating the effect of temperature on the rate of folding of DNA origami, Dietz and colleagues report that folding is a non-equilibrium process that is also highly cooperative [73]. Importantly, they show that it is possible to obtain very high yields of correctly folded complex DNA origamis in minutes by optimising folding temperature and carrying it out at a constant temperature.

Church and colleagues used the equivalent of DNA falsework, a temporary framework that is used to support construction but that can be subsequently removed, to improve the yields of their DNA box in the desired ‘closed’ conformation [74]. They introduced ‘staple’ sequences to link the two halves of the DNA box (in addition to the planned hinge and lock regions) thereby improving the yield of closed boxes from 48% to 97.5%. These staple sequences also presented toeholds that could be targeted post-assembly to remove them from the structure. A similar approach could be further developed to improve the kinetics or the pathway of folding, potentially not only improving assembly of DNA nanostructures but also enabling assembly of thermodynamically unfavourable designs.

Synthetic nucleic acids like PNA can be used for efficient removal of falsework [75] but other approaches would also be possible. For instance, if the nanostructure falsework is introduced as RNA, the need for toeholds may even be circumvented by the use of enzymes that selectively degrade RNA over DNA. The principle of DNA falsework has been successfully used to direct chemical synthesis of short sequence-defined oligomeric compounds [76,77].

Similarly, nucleic acid nanostructures are not limited to the well-defined assemblies described above but rather can form large 2D or 3D assemblies including nanoparticles and hydrogels with potential biological applications [78–80]. Karp and colleagues, using rolling circle amplification, generated DNA meshes (a 3D DNA network) containing multiple repeats of a DNA aptamer against protein tyrosine kinase-7. These meshes could be used to selectively and efficiently capture cells expressing the antigen in a microfluidic device [79]. A DNA mesh for antigen capture is compatible not only with whole cells but can also be adjusted for subcellular components or simply lysates. Together with the microfluidic potential for parallelisation, this approach could form the basis of novel panning and diagnostic platforms with considerable biomedical significance.

Nucleic acid nanoparticles were originally described by Mirkin and colleagues as gold nanoparticle cores densely coated with oligonucleotides (covalently linked to the particle by a 5′-thiol modification) or spherical nucleic acids (SNAs) [81]. A number of SNA properties are the result of the high-density oligonucleotide coating itself, including efficient cellular delivery [82]. SNAs can efficiently deliver oligonucleotides to the eukaryotic cytoplasm acting as ligands of the class A scavenger receptors and endocytosed by the caveolae-mediated pathway [83].

The high density of oligonucleotides in SNAs also affects the thermodynamic properties of annealing to complementary nucleic acid sequences – much sharper transitions between single- and double-stranded nucleic acids are observed. Despite this change, the melting profile of SNAs still correlates with the underlying coating oligo and target. Synthetic nucleic acids, such as LNA, which are known to increase duplex stability, can be introduced on the coating oligos, changing the SNA melting temperature (while keeping the sharp transition) [84,85]. Uncharged XNAs, such as PNA, have also been incorporated onto SNAs but their lack of charge resulted in unstable nanoparticles, prone to aggregation [86]. In the future, it may be possible to interface SNAs with a wide variety of XNAs to subtly alter their properties and increase thermodynamic or *in vivo* stabilities and fluorescence yields.

Hammond and colleagues demonstrated that RNA particles, made from long RNA transcripts harbouring multiple copies of hairpins generated by rolling circle transcription, could be used for cellular delivery of siRNAs. The long transcripts were reported to self-assemble into RNA particles that could be further condensed with polyamines creating a nucleic acid nanoparticle that could be efficiently uptaken and processed by cells in culture, delivering an estimated 10^6^ copies of the siRNA hairpin per cell [80]. A similar increased cell penetration has been reported for DNA nanotubes generated partly by rolling circle amplification [87]. It remains to be established whether efficient delivery to cells in culture translates into efficient delivery *in vivo* but in both cases, the reports highlight that large or at least structured nucleic acid molecules may be able to enter cells efficiently via lysosomal uptake pathways [88], potentially exploiting the same internalisation mechanism of SNAs.

## Concluding remarks and future perspectives

**Figure 2:**
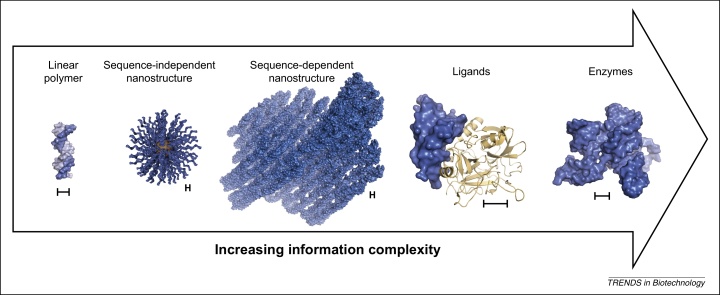
Information complexity in nucleic acid materials. As a material, nucleic acids encode more information than what is stored in the aperiodic polymer [110–113]. Topological arrangements that deviate from the linear molecule, such as nanoparticles like spherical nucleic acids [78] or precise structures such as DNA origami [114] [Protein Data Bank (PDB): 2YMF, 2YMG, 2YMH, 2YMI, and 2YMR] increase the complexity of the stored information and can lead to novel physicochemical properties, such as spherical nucleic acid (SNA) efficient cellular uptake [83,115] or nucleic acid structures that function as pores in lipid bilayers [62,63]. Even higher information complexity can be obtained through the isolation of individual sequences capable of high affinity and specific binding to ligands (aptamers) such as an aptamer against thrombin (PDB: 3DD2) [116] or with catalytic activity such as an RNA ligase ribozyme (PDB: 3HHN) [117]. Structures are drawn to scale with the bar representing approximately 12.5 Å.

Nucleic acids have shown to be a remarkably versatile source of novel materials and nanodevices. Design and evolution combined with the sub-angstrom resolution offered by nucleic acid scaffolds may enable not only the assembly of intricate structures but also confer them with properties such as tight ligand binding, catalytic activity, and different conformational states (Figure 2).

Chemical modification of nucleic acids, and in particular wholesale replacement of the natural framework with synthetic genetic polymers (XNAs) capable of evolution, has the potential to create novel structures and devices as well as greatly extend their functionalities – for instance, generating XNA devices that are stable *in vivo* and that can be used in diagnostic applications capitalizing on fluorescence or catalytic properties. Given the limited chemical and biological stability of DNA and RNA, it may also be possible to use DNA and RNA nanostructures as formwork (moulds) for the synthesis/condensation and assembly of other polymer-based structures and materials.

Modifications that extend the chemical repertoire available to nucleic acids will also enable a wider range of properties to be accessed, depending on the chemical functionality introduced and on the density of the substitution. Ongoing work in our labs to expand the chemistry that is compatible with enzymatic synthesis and evolution will add to the ever growing palette of XNAs available for the construction of XNA nanostructures and devices enabling novel functions and improved performance in challenging environments and conditions.
